# For Hospital Workers

**Published:** 1914-04-04

**Authors:** 


### For Hospital Workers.

It is our purpose to make each number of The Hospital of practical use and interest to hospital workers in every department of institutional service. We could wish that the secretaries and matrons would direct the attention of their staff in the various departments to the *Institutional Worker* section of The Hospital. In that section our aim is to exchange information with all institutional workers. A reference to its pages will show that recently it has contained practical information on contracts for supplies; ward furniture, with some useful illustrations of new types of equipment; original designs for coal bunkers, with full descriptions and illustrations of more than one kind; the comparative values of meat, with full details, and a comparison of the relative nutritive qualities of foreign and English meat; the duties and plan of work of the male servants in a hospital; the service of food in three different types of nursing institutions; and the system of filing out-patient case papers. Will the more practical of our readers keep an eye on this section and help us with a little personal service by making it known to their staff? The columns for Questions Invited and Questions To Be Answered present a great opportunity. In particular we would direct the attention of medical superintendents and others to the important question which appears this week on page 2 of the *Institutional Worker* on baths for continuous immersion, and our readers are invited to give their experience.

